# THE POTENTIAL AND PITFALLS OF GAMIFICATION TO SUPPORT OLDER ADULTS' ADHERENCE TO HEALTHCARE INTERVENTIONS

**DOI:** 10.1093/geroni/igz038.125

**Published:** 2019-11-08

**Authors:** Walter R Boot, Neil Charness

**Affiliations:** Florida State University, Tallahassee, Florida, United States

## Abstract

The addition of video game-like elements to non-game activities, known as gamification, holds promise with respect to encouraging engagement with, and adherence to, health behaviors and healthcare interventions. Elements of gamification include the introduction of points systems, leaderboards, achievement badges, stories and themes, rewards, progress tracking, and challenges. However, a lack of enthusiasm for, and experience with, video game play by older adults has important implications for the effectiveness of these techniques across the lifespan. Specifically, the age-related “digital divide” must be considered before applying these approaches to improving the wellbeing, cognition, and health of older adults. This talk will build on the body of research conducted by the Center for Research and Education on Aging and Technology Enhancement (CREATE) focused on gaming and interventions to present best practice guidelines for implementing gamification with older adults.

